# Objectively Measured Physical Activity Increases Only in Males During a Summer Camp for Obese Children

**DOI:** 10.3389/fspor.2021.624449

**Published:** 2021-03-25

**Authors:** Pascal Izzicupo, Andrea Di Blasio, Andrea Di Credico, Barbara Ghinassi, Laura Capranica, Giorgio Napolitano, Angela Di Baldassarre, Elisabetta Modestini, Mario Di Pietro

**Affiliations:** ^1^Department of Medicine and Aging Sciences, University “G. D'Annunzio” of Chieti-Pescara, Chieti, Italy; ^2^Department of Movement, Human and Health Sciences, University of Rome Foro Italico, Rome, Italy; ^3^Pediatrics Unit, Hospital of Atri, Atri, Italy

**Keywords:** childhood obesity, physical activity, summer camp, accelerometry, sedentary behavior, activitystat, children

## Abstract

Childhood obesity is a major public health challenge. Summer camps for children with obesity represent an alternative setting to improve eating and physical activity habits. Here we evaluated if the participation in the camp improves objectively measured physical activity and sedentary behavior and whether there are differences between male and female participants. Twenty-eight children, 13 males and 15 females (body mass index >97° centile, weight excess >30%, Tanner stage I), agreed to participate in an 8-day camp. During the summer camp, children participated in sports-like games and outdoor activities for at least 3 h a day, and the school-camp staff also provided a theoretical nutritional learning plan. Accelerometry-derived physical activity was measured through the SenseWear Mini Armband during a week at home and during the camp experience. Before camping, the participants were far above the minimum daily values of moderate- to vigorous-intensity physical activity (MVPA) to be considered sufficiently active (≥60 min/day), but male participants were more active than females (MVPA: 186.2 ± 94.2, 111.0 ± 64.7; *P* = 0.020). Male participants increased their MVPA (234.3 ± 114.8, *P* = 0.020), whereas females not (111.9 ± 52.9, *P* = 0.020). No difference emerged for the sedentary behavior either before or during the camp. This study suggests that participation in a summer camp for obese children can determine different responses in physical activity levels, depending on the sex of young participants. Thus, summer camps for obese children should put particular attention on female participants, besides reducing sedentary behavior in both males and females.

## Introduction

Childhood obesity is a major public health challenge, and several studies suggest that obesity is continuously increasing among the young population in many, but not all countries worldwide. These international data indicate that in the vast majority of high-income and upper middle-income countries, a stark difference in the prevalence of obesity by sex exists, with boys showing a greater obesity prevalence than girls (Shah et al., 2020). The number of school-aged children and adolescents facing obesity is predicted to rise from 150 million worldwide to more than 250 million by 2030 (Shah et al., 2020), putting a massive burden on healthcare systems with the aging population (Hruby and Hu, 2015). Indeed, obesity implications relate not only to the present health status of younger people. Obese children are at higher risk of developing adult obesity and related complications later in life, such as atherosclerosis, type 2 diabetes mellitus, and cardiovascular diseases (Freedman et al., 2007; Haines et al., 2007; Gordon-Larsen et al., 2010). Therefore, the treatment of obesity plays a central role in public health. However, traditional interventions of obese children and adolescents with dietary restrictions associated or not with physical activity programs often fail (Mauro et al., 2008). The adoption of multidisciplinary and intensive protocols could overcome the limitations of traditional ones. In this context, camps for obese children represent a useful instrument for nutritional and lifestyle re-education, improving short- and long-term eating, and physical activity habits (Di Pietro et al., 2004; McCarty et al., 2012; Barnett et al., 2018). Previous physical activity is a key determinant of actual physical activity in children (Condello et al., 2017), and there is evidence that an 8-day summer camp is enough to determine positive behavioral changes toward physical activity in the long term (Di Pietro et al., 2004). A previous study on asthmatic children indicated that camp experience improves intentional health behavior adoption (Lin et al., 2008) according to the Stages of Change Model (SCM). The SCM describes this process of health behavior change as a gradual process encompassing different stages, also influenced by self-efficacy and social support (Marcus et al., 1992; Wallace et al., 2000). Moreover, important determinants of children's physical activity, such as time spent outdoors (Puggina et al., 2018) or having a companion for physical activity (Jaeschke et al., 2017), might be prompted by camping experience.

The relationship between accelerometer-determined physical activity and accurate body composition measures in children is well-known (Jiménez-Pavón et al., 2010). Moreover, while obese children spend more time engaged in sedentary behavior (Maffeis et al., 1996), compared with non-obese children, no differences emerge in total lean body mass–adjusted physical activity energy expenditure assessed through the doubly labeled water technique, despite lower accelerometer-derived physical activity levels (Ekelund et al., 2002). Thus, it is important to add insights into the field of childhood obesity and physical activity to adopt the best countermeasures. While most studies focused on diet or investigated self-reported physical activity, there is a lack of objective data regarding the physical activity performed on camps for obese children. Furthermore, the recent evidence of sex/gender-related difference in childhood obesity prevalence, together with findings suggesting a difference between boys and girls in physical activity levels (Trost et al., 2002; Sherar et al., 2007; Munakata et al., 2010; Hallal et al., 2012; Pearce et al., 2012; Telford et al., 2016; Aleksovska et al., 2019), highlights the importance of considering the role of sex in treating obesity in children and adolescents. Therefore, here we present an experience on the effect of a short-term summer camp in obese children in Italy, aiming to evaluate if the participation in the camp improves daily physical activity and sedentary behavior and whether there are differences between male and female participants.

## Materials and Methods

### Participants

Twenty-eight prepubertal children from a rural area in central Italy, 13 males [9.8 ± 1.7 years, body mass index (BMI) 27.0 ± 3.7 kg/m^2^] and 15 females (8.5 ± 1.8 years, BMI 26.9 ± 4.0 kg/m^2^), attending the outpatient clinic, agreed to participate. To be selected for participation in the study, young patients had to fulfill the following inclusion criteria: not participate in sports activities from at least 1 year, BMI >97° centile, weight excess >30%, and Tanner stage I. For each participant, parents gave informed consent and accompanied their children during the 1st day, on a weekday, and on the last day of the camping. The Ethics Committee of the “G. D'Annunzio” University of Chieti–Pescara approved this study.

### Procedure

Weight, height, and objective physical activity were assessed about a week before and during the participation in the camp. In particular, participants wore a triaxial multisensory device (SenseWear Mini Armband; BodyMedia, Inc., Pittsburgh, PA) during the week before and during the camping week. Weight was assessed three times when the device was delivered at the beginning and the end of camp for obese children. School camp for obese children took place during the summer holidays in a seaside resort and lasted 8 days. The camp team comprised two pediatricians, a dietitian, a psychologist, two sport scientists, a schoolteacher, and a nurse. During camping, participants had five slightly hypocaloric meals a day (basal metabolism × 1.2) and participated in sports-like games and outdoor activities for at least 3 h a day. In particular, games were chosen and designed to allow participants to be gratified by participation despite their weight excess and comprised games inspired to judo, wrestling, and other combat sports; Latin sport dances; throwing sports; and orienteering. In particular, combat sports and sport dances were chosen to satisfy both male and female preferences. The summer camp staff also provided a theoretical nutritional learning plan, whereas outdoor activities included educational visits to the farm and the surrounding natural environment.

### Anthropometry

A first-level anthropometrist of the International Society for the Advancement of Kinanthropometry carried out the body measurements of the participants in their fasting condition. Body mass and stretched stature were measured to the nearest 0.1 kg and 0.1 cm, respectively, with the participants dressed in light clothing and without shoes, using a stadiometer with a balanced-beam scale (Seca 220; Seca, Hamburg, Germany). The participant BMIs were calculated as body mass/stature^2^ (kg/m^2^). The evaluation of the weight status was obtained from BMI, according to Cacciari et al. (2006).

### Physical Activity and Sedentary Behavior Measurement

Daily physical activity and sedentary behavior were measured under free-living conditions for 5 consecutive days, including 3 weekdays and at least 1 weekend day, using SenseWear Mini Armbands (Welk et al., 2007; Scheers et al., 2013; Wetten et al., 2014). The participants wore their monitors all through the 5 measurement days, except while bathing. No raining days were present among the recorded periods. The wear time criteria were at least 600 min/day with at least 3 valid weekdays and a valid weekend day. It has been shown that 3 weekdays of recording using the SenseWear Pro3 Armband were enough to achieve a reliability of 0.80 for sedentary behavior, light-intensity physical activity (LIPA), moderate-intensity physical activity (MPA), physical activity level, and energy expenditure (Scheers et al., 2012). The SenseWear Mini Armband relies on the same technology but mounts a 3-axis accelerometer instead of the 2-axis version in the SenseWear Pro3 (Johannsen et al., 2010). The internal algorithms are slightly different between the monitors. However, the SenseWear Mini Armband showed slightly better performance over the SenseWear Pro3 (Johannsen et al., 2010). The SenseWear Mini Armband integrated the information gathered by the 3-axis accelerometers and sensors (i.e., skin and near-body temperature, heat flux, galvanic skin response) with the sex, age, stature, weight, smoking status, and handedness of the user, using the SenseWear Professional 8.0 software (BodyMedia, Pittsburgh, USA). It has been shown that combining accelerometry with physiological parameters can improve measurement accuracy (Welk et al., 2007; Johannsen et al., 2010; Wetten et al., 2014). The SenseWear Mini Armband provided extensive information about wear time, daily physical activity (e.g., intensity, number of daily steps, energy expenditure), and other behaviors such as sleep and sedentary behavior. The SenseWear Mini Armband was worn on the left arm, over the triceps, as it has been shown able to furnish reliable recording compared with other placements (i.e., wrist, hip, ankle), and in respect to both other devices and placements (Wetten et al., 2014), in both healthy and pathological conditions (Welk et al., 2007; Scheers et al., 2013), according to the manufacturer's validation.

From the recorded data, the focus in the present study was on time spent on physical activity according to three intensity levels: an intensity >3 METs and ≤6 METs (i.e., MPA); an intensity >6 METs and ≤9 METs (i.e., vigorous-intensity physical activity; VIPA); and an intensity >9 METs (i.e., very vigorous-intensity physical activity). The total time spent above 3 METs was considered as moderate- to vigorous-intensity physical activity (MVPA). Besides, sedentary behavior and the time spent in LIPAs were considered. As SenseWear Mini Armband cannot distinguish between sitting and standing, sedentary behavior was considered as the whole time spent in physical activities with an intensity ≤1.5 METs, excluding nocturnal sleeping. The LIPA was the time spent in physical activities >1.5 METs and ≤3 METs. The numbers of daily bouts of sedentary behavior that lasted <6 consecutive min (SB_sporadic_), between 6 and 10 min (SB_medium_), 11 and 30 min (SB_long_), and longer than 30 min (SB_verylong_) were calculated using a specifically written application (Izzicupo et al., 2020).

### Statistical Analysis

Data were analyzed using the Statistical Package for the Social Science, version 24.0 (SPSS Inc., Chicago, IL) and initially tested for normality with the Shapiro–Wilk statistic and presented as mean ± SD. An *a priori* level of significance was set at *p* < 0.05. As data were not normally distributed, the Mann–Whitney *U*-test was applied to investigate the effect of the independent variable sex on the dependent variables weight, BMI, and objectively measured physical activity and sedentary behavior. Wilcoxon signed-rank test was applied to investigate differences in weight, BMI, and objectively measured physical activity and sedentary behavior before and after the participation at the campus in both male and female participants.

## Results

### Physical Activity and Sedentary Behavior Before Camping

Before camping, the participants were far above the minimum daily values of MVPA (≥60 min/day) to be considered sufficiently active but spent a considerable amount of time in sedentary pursuits (≥240 min/day). Most of the day was spent in LIPAs, and more than two episodes per day of sedentary behavior exceeding 30 min took place. Despite the high levels of physical activity, male participants were more active than females in MPA, VPA, and MVPA (Table 1).

**Table 1:** Physical activity and sedentary behavior before camping.

	**Females (*n* = 15)**	**Males (*n* = 13)**	***P***
Steps (*n*.)	11,880 ± 3,199	14,516 ± 5,275	0.205
METs	1.77 ± 0.38	2.00 ± 0.35	0.147
SB (min)	280.6 ± 289.4	309.3 ± 259.4	0.982
LIPA (min)	642.2 ± 333.4	543.0 ± 272.3	0.420
MPA (min)	105.3 ± 60.0	165.3 ± 82.8*	0.045
VPA (min)	5.7 ± 6.3	20.9 ± 23.6*	0.025
MVPA (min)	111.0 ± 64.7	186.2 ± 94.2*	0.020
SB_sporadic_ (*n*.)	38.9 ± 16.9	44.9 ± 25.1	0.629
SB_medium_ (*n*.)	6.2 ± 4.7	7.7 ± 5.2	0.394
SB_long_ (*n*.)	4.8 ± 5.8	5.8 ± 5.8	0.854
SB_verylong_ (*n*.)	2.4 ± 4.1	1.9 ± 2.5	0.522

METs, metabolic equivalent of the task; SB, sedentary behavior; LIPA, light-intensity physical activity; MPA, moderate-intensity physical activity; VPA, vigorous-intensity physical activity; MVPA, moderate- to vigorous-intensity physical activity; SB_sporadic_, sedentary behavior shorter than 6 min; SB_medium_, sedentary behavior between 6 and 10 min; SB_long_, sedentary behavior between 11 and 30 min; SB_verylong_, sedentary behavior episodes longer than 30 min. *Significant difference between male and female participants.

### Physical Activity and Sedentary Behavior During Camping

**Figure 1:**
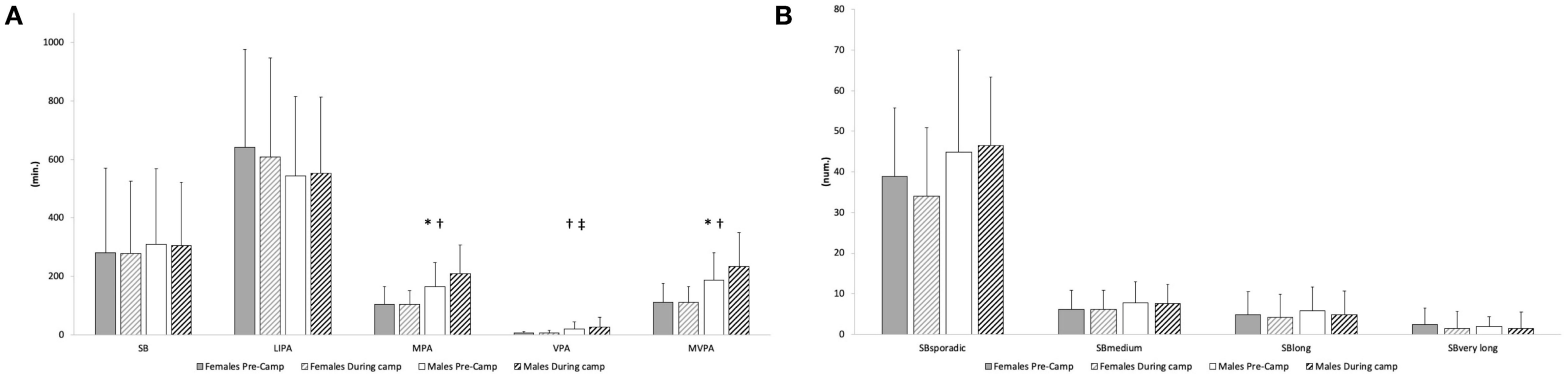
**(A)** Physical activity and sedentary behavior before and during camping. **(B)** sedentary episodes of different length before and during camping. SB, sedentary behavior; LIPA, light-intensity physical activity; MPA, moderate-intensity physical activity; VPA, vigorous-intensity physical activity; MVPA, moderate- to vigorous-intensity intensity physical activity; SB_sporadic_, sedentary behavior shorter than 6 min; SB_medium_, sedentary behavior between 6 and 10 min; SB_long_, sedentary behavior between 11 and 30 min; SB_verylong_, sedentary behavior episodes longer than 30 min. *Significant difference between male and female participants before camping. ^†^Significant increase in male participants during the camp. ^‡^Significant difference between male and female participants during the camp.

During the participation in the camp, male participants increased their MPA and MVPA, whereas females maintained unchanged their physical activity levels. As a consequence, MPA, VPA, and MVPA were higher in male participants than in females during the camp. No difference emerged for sedentary behavior, LIPA, and for the sedentary behavior episodes of any investigated lengths (Figure 1). Furthermore, steps per day and average METs per day were also higher in males than in females (16,472 ± 3,774, 12,058 ± 2,170; and 2.10 ± 0.41, 1.76 ± 0.35, respectively). No significant reduction in weight and BMI was observed at the end of the camp.

## Discussion

In this study, we found that the camp experience in children with obesity determines different changes in physical activity levels between males and females. In particular, whereas male children increased MPA and MVPA during the camp compared with their levels before camping, females unchanged their habits in terms of physical behaviors. Furthermore, male participants showed higher MPA, VPA, and MVPA, even before participating in the camp. Additionally, there was no difference in sedentary behavior between males and females, and the camp participation did not determine changes. These results are consistent with persistent findings in the literature indicating that female children are less active than males (Trost et al., 2002; Sherar et al., 2007; Munakata et al., 2010; Hallal et al., 2012; Pearce et al., 2012; Telford et al., 2016; Aleksovska et al., 2019). Previous studies indicate several potential gender factors affecting female children and adolescents' physical activity levels, including lower perceived enjoyment when taking part in physical education (Cairney et al., 2012) and less social support to engage in physical activity (Edwardson et al., 2013), which could, in turn, lead them to participate less in organized sports (Vella et al., 2014). Furthermore, lower physical activity levels in girls may be related to maturing at an earlier chronological age, suggesting that biological reasons may also contribute to the sex difference in physical activity (Sherar et al., 2007; Wickel et al., 2009). Independently from sex- and gender-related factors, the measured levels of physical activity are far above the levels reported for the general pediatric population and obese children. Although surprising, several factors may have determined such a high level of activity, particularly the summer season and the rural location. Seasonal variations in children's physical activity have been previously reported, with a peak during June and higher levels for those living in a rural area, particularly during the weekend days (Atkin et al., 2016). Other factors, such as family income and cultural influences, might have further affected our sample's physical activity levels. However, their collection was beyond the scope of the present investigation. Finally, the device used in the present study for physical activity assessment might return higher values than others, although validated in children for energy expenditure in semistructured activities settings (Lee et al., 2016). Indeed, physical activity levels very close to the ones of the present study (boys: 179 ± 90 min/day; girls: 136 ± 59 min/day) were already reported in a previous investigation using the SenseWear Armband (Soric and Misigoj-Durakovic, 2010).

Previous studies showed that male children dedicate more time to sedentary pursuits such as television watching (Munakata et al., 2010), but no difference emerged between the two gender groups in sedentary behaviors, in the present study. There is evidence that screen time (time spent in visual media activities on screen devices, including watching television or videos, playing games, video chatting, searching the Internet, and reading or writing on a computer, tablet, or smartphone for entertainment purposes) can be more deleterious than other types of sedentary behavior, such as passive transportation, school time, and studying, from both a health and behavioral perspective. Screen time may affect sleep time and quality, as well as eating disorders, aggression, sexual behavior, substance abuse, and academic difficulties (Strasburger et al., 2010; Jones et al., 2019). However, as we used objective measurements, we cannot distinguish for diverse sedentary activities.

Physical activity is a potent protective factor against the development of childhood obesity (Chung et al., 2012; Di Blasio et al., 2016). On the other hand, the decline in physical activity during adolescence increases the risk of becoming obese in adulthood (Pietiläinen et al., 2008). These findings seem partially in contrast with the higher obesity prevalence in males comparing to females. While it is true that the latter are less active and that physical activity safeguards against childhood obesity, it should be expected that the prevalence of obesity will be higher in girls than in boys. Furthermore, in the present study, both males and females were far above the minimum daily values of MVPA to be considered sufficiently active, even though they were obese. However, younger obese children generally meet the daily recommendations for physical activity (Chung et al., 2012), and the results of the present study are consistent with this evidence (Table 1). Older children, regardless of weight status, did not meet physical activity recommendations, especially girls (Trost et al., 2002; Sherar et al., 2007; Chung et al., 2012; Aleksovska et al., 2019). In particular, the decline in physical activity in females may occur earlier because of anticipated sexual maturity (Sherar et al., 2007; Wickel et al., 2009) with respect to males. Considering both the decline in physical activity occurring with aging and the central role of an active lifestyle in preventing body weight gains, multidisciplinary treatments of obesity, including physical activity, have been extensively designed and investigated.

Camps for obese children can improve short- and long-term eating and physical activity habits (Di Pietro et al., 2004; McCarty et al., 2012). However, in the present study, the camp experience did not improve physical activity levels in female children, while it was effective in males. Participants took part equally in the organized outdoor activities and sport-like games. Furthermore, camp activities were designed to satisfy both males' and females' preferences. Notwithstanding, lower perceived enjoyment when taking part in physical education in girls (Cairney et al., 2012) and other psychosocial factors might have affected the female involvement levels during the organized activities, resulting in lower measured physical activity. Alternatively, the source of the difference found between male and female children might be due to the spontaneous physical activity during the remaining part of the day. It is possible that female campers compensated for physical activity performed outdoor and during games, reducing the activity in the remaining part of the day. There is still debate in the scientific community about compensation mechanisms in physical activity after its increase in a part or during the whole day. The “activitystat” and “energystat” hypotheses sustain the existence of innate set points that control physical activity or energy expenditure over time (Rowland, 1998; Gomersall et al., 2013). They are hypothesized to regulate physical activity and energy expenditure via homeostatic feedback processes (Wilkin, 2011). As such, increases in physical activity, sedentary time, or energy expenditure in one part of the day are supposed to result in decreases in physical activity, sedentary time, or energy expenditure, respectively, in another part of the day (Rowland, 1998; Rowlands, 2009), as well as in the subsequent days (Ridgers et al., 2018).

Several studies reported findings confirming or confuting such hypotheses. It was also demonstrated that, in some people, an additive effect could follow the introduction of an exercise program, making them even more active at other times (activity synergy) (Goodman et al., 2011; Di Blasio et al., 2012, 2014, 2018; Izzicupo et al., 2013a). On the other hand, despite the evidence that the genotype could partly influence training adaptation and spontaneous activity (Izzicupo et al., 2010, 2013b; Lightfoot et al., 2018), physical inactivity leads to obesity independent of genetic factors, as demonstrated in a rare group of monozygotic twin pairs discordant for obesity in young adulthood (Pietiläinen et al., 2008). Our study indicates that females perform less physical activity than males during a summer camp for obese children. This result may be due to a lower involvement in structured activities, the reduction of spontaneous physical activity during the remaining part of the day, or both. Considering the difference in physical activity during the camp between male and female children observed in this study, it is also possible that both physical activity compensation and synergy can occur in obesity treatment, depending on the sex of the participants. However, determining if this effect is due to an innate, biological set point or by gender stereotypes was beyond the aims of the present study.

Participants' sedentary time did not change during the camp experience, despite increased MPA, VPA, and MVPA in males. This result suggests that the participant replaced LIPA and sleep time instead of sedentary behavior to perform MVPA. Ideally, an intervention should increase physical activity and reduce sedentary time, especially addressing screen time (Ricci et al., 2020), leaving sleep unaltered, or improving it if inadequate. Thus, understanding how to avoid compensation and favoring activity synergy is a priority for future research that can help to design obesity treatment in children in consideration of sex/gender factors.

This study has a series of limitations. First, the sample size was small, and data not normally distributed, making us lean toward non-parametric analysis. However, the sample was slightly larger than the minimum required for medium effect size (*n* = 24). Second, detailed data collection was performed only for objectively measured physical activity and sedentary behavior, without any other biomarker or social and environmental factors explaining current findings. Furthermore, relying only on the objective measurement of physical activity, we did not detect important aspects through the use of questionnaires or diaries that would have allowed us to evaluate the physical activity and sedentary behaviors domains, as well as to take a deeper look at the physical activity levels actually carried out during the structured activities. Third, sex/gender factors were not systematically reported, although camp activities' design considered them. However, the present study also has strengths. To our knowledge, this is the first study describing objectively measured physical activity and sedentary behavior in obese children during a camp experience. Furthermore, the intensive nature of a camp for obese children allows introducing significant changes in participants' lifestyles, which are challenging to obtain with home-based programs or sports participation, especially in terms of quantity of physical activity. This aspect is particularly relevant for investigating the compensation events of physical activity, and the homogeneity of the sample, composed only of obese children who did not take part in sports activities, reinforces our results.

## Conclusions

The present study indicates that participation in a summer camp for obese children can determine different responses in spontaneous physical activity, depending on the sex of young participants. Camps for obese children represent a useful instrument for long-term nutritional and lifestyle re-education. However, our study demonstrates that sedentary behavior remains unchanged in the short term, regardless of sex, and female obese children do not improve physical activity. Thus, future studies should (i) address explanatory variables for compensation effect in females during camps participation and (ii) manipulate daily activities trying to reduce sedentary time to provide insights useful for a better designing intensive period of treatment of obesity through training camps.

## Practical Application

Childhood obesity is a major public health challenge, but the traditional treatment of obese children and adolescents with dietary restrictions associated or not with physical activity programs often fails. Thus, camps for obese children have been proposed for nutritional and lifestyle intensive re-education. Sports-like games and outdoor activities are often part of the summer camp programs and are supposed to increase children's physical activity levels. However, several studies indicate that increases in physical activity in one part of the day may result in subsequent, compensative decreases in physical activity in other parts of the day. From a practical point of view, this study suggests that during summer camps for obese children, physical activity and sedentary behavior should be monitored through objective measurement to quantify compensation in physical activity and sedentary behavior. Questionnaires can also be useful to determine which kind of specific sedentary behavior is performed by children. Strategies for addressing sedentary behavior should be designed, as proposed sport-like games and outdoor activities do not decrease sedentary time in the remaining part of the day. Finally, girls need special attention during leisure time, as they compensate for structured physical activities in the remaining part of the day.

## Data Availability Statement

The raw data supporting the conclusions of this article will be made available by the authors, without undue reservation.

## Ethics Statement

The studies involving human participants were reviewed and approved by the ethics committee of the University of Studies G. D'Annunzio of Chieti - Pescara. Written informed consent to participate in this study was provided by the participants' legal guardian/next of kin.

## Author Contributions

PI, ADBl, and MD: conceptualization and design of the study. PI, AD, and BG: methodology. PI and AD: formal analysis. PI, ADBl, EM, AD, and BG: data collection. AD, BG, and LC: data curation. PI, AD, and ADBl: writing—original draft preparation. BG, GN, LC, and EM: visualization. PI, LC, MD, GN, and ADBa: supervision and editing. ADBa: funding acquisition. All authors have read and agreed to the published version of the manuscript.

## Conflict of Interest

The authors declare that the research was conducted in the absence of any commercial or financial relationships that could be construed as a potential conflict of interest.
